# Oral administration of MCT oil reduces anxiety-like behavior and is associated with differences in serum BDNF responses in a rat model of PTSD

**DOI:** 10.1038/s41598-026-46116-6

**Published:** 2026-04-06

**Authors:** Daisuke Yoshioka, Takehiko Yamanashi, Koji Komatsu, Naofumi Kajitani, Chika Ushida, Miyu Matsumi, Moyu Nakamoto, Kaori Adachi, Ryoichi Matsuo, Akihiko Miura, Tsuyoshi Nishiguchi, Masaaki Iwata

**Affiliations:** 1 Department of Neuropsychiatry, Faculty of Medicine, Tottori University, 86 Nishi-cho, Yonago, 683-8503 Tottori Japan; 2 Organization for Research Initiative and Promotion, Tottori University, Tottori, Japan

**Keywords:** Post-traumatic stress disorder, Medium-chain triglyceride, β-hydroxybutyrate, Brain-derived neurotrophic factor, Anxiety-like behavior, Diseases, Neuroscience, Physiology

## Abstract

Post-traumatic stress disorder (PTSD) is characterized by neuroinflammatory and neurotrophic dysregulation, and effective interventions remain limited. Medium-chain triglyceride (MCT) oil, a dietary source of ketone bodies, may offer a practical means to elevate circulating β-hydroxybutyrate (BHB), a molecule with reported anti-inflammatory and neuroprotective properties. This study investigated whether oral administration of MCT oil modulates behavioral and molecular stress responses in a single prolonged stress (SPS) rat model of PTSD. Repeated oral MCT administration significantly increased circulating BHB levels and reduced anxiety-like behavior in the elevated plus maze, without affecting locomotor activity. Under acute restraint stress, serum brain-derived neurotrophic factor (BDNF) levels were significantly lower in MCT-treated rats than in long-chain triglyceride (LCT)-treated controls. Inflammatory cytokine expression remained unchanged in serum and brain tissue. These findings suggest that oral MCT oil attenuates anxiety-like behavior under SPS conditions and is associated with differences in serum BDNF responses during acute stress. Therefore, MCT oil may represent a feasible dietary approach for influencing stress-related behavioral responses.

**Supplementary Information:**

The online version contains supplementary material available at 10.1038/s41598-026-46116-6.

## Introduction

Post-traumatic stress disorder (PTSD) is a debilitating psychiatric condition that develops after exposure to severe trauma, such as war, disasters, or interpersonal violence. It is characterized by four core symptom clusters: intrusive re-experiencing, persistent avoidance, negative alterations in cognition and mood, and hyperarousal. Additionally, PTSD is frequently associated with anxiety, mood, and substance use disorders^1^. Although selective serotonin reuptake inhibitors (SSRIs) and psychological therapies are standard treatments, 20–50% of patients show poor or no response^2^. Therefore, the development of novel therapeutic strategies is crucial.

Recent studies have suggested that the pathophysiology of PTSD involves complex interactions among brain regions, including the hippocampus, amygdala, and prefrontal cortex (PFC)^3^. Dysregulation of neuroinflammatory processes^4–6^ and neurotrophic factors^7^ has also been implicated as an important mechanism underlying this disorder. Preclinical studies have shown that hippocampal mRNA levels of interleukin-1beta (IL-1β) and tumor necrosis factor-alpha (TNF-α) are increased in a rat model of PTSD, and that substances ameliorating PTSD-related behaviors attenuate the elevation of hippocampal inflammatory cytokine mRNA^8,9^. Furthermore, a recent meta-analysis reported that some inflammation-related biomarkers, including TNF-α and IL-1β, are higher in individuals with PTSD than in healthy controls^4,6^. In addition to neuroinflammation, brain-derived neurotrophic factor (BDNF) —a neurotrophin involved in neuronal survival and synaptic plasticity—has been implicated in PTSD. Rodent models of PTSD exhibit decreased BDNF levels, particularly in the hippocampus, PFC and amygdala^10,11^. Similarly, clinical studies have reported reduced serum and plasma BDNF levels in patients with PTSD^12^. These findings suggest that substances targeting these pathological pathways may represent novel treatment candidates for PTSD.

β-hydroxybutyrate (BHB) is a ketone body primarily known for supporting mammalian survival during energy-deficit states by serving as an alternative energy substrate to glucose^13,14^. Beyond its principal metabolic role, BHB reduces the release of inflammatory cytokines by inhibiting activation of the NOD-like receptor family pyrin domain-containing 3 (NLRP3) inflammasome^15^. Furthermore, experimental studies have demonstrated that BHB increases BDNF expression in hippocampal and cortical neurons, in vitro and in vivo, supporting the notion that BHB functions as a signaling molecule informing neurons of metabolic state shifts^16,17^.

We previously demonstrated that BHB exerts antidepressant effects in rodent models of depression by inhibiting neuroinflammation^18,19^. Additionally, our laboratory has shown that BHB administration attenuates anxiety-like behavior and suppresses inflammatory cytokines in a single prolonged stress (SPS)-induced rat model of PTSD^20^. However, in that study, BHB was administered subcutaneously, which poses practical limitations for clinical application.

Medium-chain triglycerides (MCTs) —composed of fatty acids with carbon chain lengths of C6–C12^21^—are naturally present in coconut oil, dairy products, and human breast milk^22–24^. Unlike long-chain triglycerides (LCTs), which require specific membrane transporters and carnitine-dependent mitochondrial import, medium-chain fatty acids (MCFAs) derived from MCTs are absorbed rapidly via the portal vein and directly transported into mitochondria without the need for a carnitine shuttle^25^. In the liver, MCFAs undergo β-oxidation to generate acetyl-CoA, which, when exceeding the capacity of the citric acid cycle, is converted into ketone bodies—primarily BHB, and to a lesser extent acetoacetate. These ketone bodies are transported to the brain, muscles, and other peripheral organs^26,27^. This rapid conversion makes MCT oil an efficient oral precursor for increasing systemic BHB levels and offers a practical alternative to parenteral administration.

Based on these characteristics, we hypothesized that oral supplementation with MCT oil could provide a feasible and noninvasive strategy to elevate circulating BHB levels and exert therapeutic effects similar to those observed with subcutaneous BHB administration. Therefore, we investigated whether daily oral administration of MCT oil attenuates anxiety-like behavior in rats subjected to the SPS paradigm. We also examined whether these behavioral effects are associated with changes in neuroinflammatory and neurotrophic factors.

## Results

### Time course of blood BHB levels following oral MCT oil administration

**Fig. 1:**
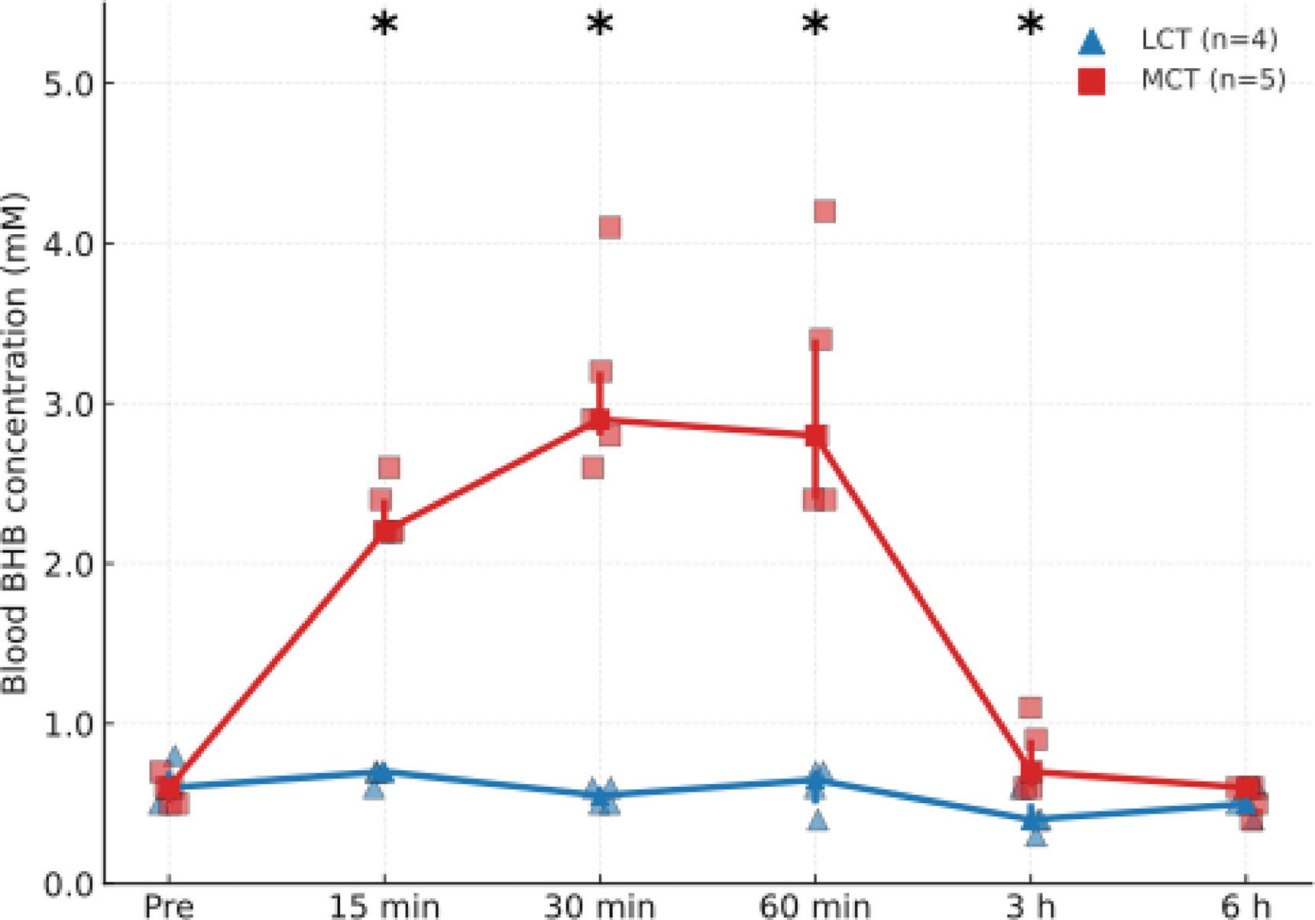
Time course of blood BHB concentrations following oral MCT oil administration. Rats were orally administered 0.8 mL of MCT or LCT oil, and blood samples were collected at baseline and at 15 min, 30 min, 1 h, 3 h, and 6 h post-administration. BHB concentrations were significantly higher in the MCT group than in the LCT group at 15 min (median [IQR]; LCT: 0.70 [0.67–0.70], MCT: 2.20 [2.20–2.40], *p* = 0.016), 30 min (LCT: 0.55 [0.50–0.60], MCT: 2.90 [2.80–3.20], *p* = 0.019), 1 h (LCT: 0.65 [0.55–0.70], MCT: 2.80 [2.40–3.40], *p* = 0.019), and 3 h (LCT: 0.40 [0.38–0.45], MCT: 0.70 [0.60–0.90], *p* = 0.033). No significant differences were observed at baseline (*p* = 0.70) or at 6 h (*p* = 0.51). These results indicate that oral MCT oil rapidly and transiently elevated circulating BHB levels compared with LCT oil. Data are shown as line plots indicating the median and IQR. The Mann–Whitney U test was used for statistical comparisons. Sample sizes: MCT group, *n* = 5; LCT group, *n* = 4. BHB, β-hydroxybutyrate; MCT, medium-chain triglyceride; LCT, long-chain triglyceride; IQR, interquartile range; *p < 0.05.

To evaluate the time-dependent effects of MCT oil on blood BHB concentrations, rats were administered a single dose of MCT or LCT oil (0.8 mL) via oral gavage, and blood BHB levels were measured at 0 (pre-administration), 15, 30, and 60 min, and at 3 and 6 h post-administration. Mann–Whitney U tests were performed at each time point to compare the two groups. Significant differences in BHB levels were observed at 15 min (*p* = 0.016), 30 min (*p* = 0.019), 60 min (*p* = 0.019), and 3 h (*p* = 0.033), with the MCT group exhibiting higher BHB concentrations than the LCT group. No significant differences were detected at baseline or at 6 h (Fig. 1). The MCT group consisted of 5 rats, and the LCT group consisted of 4 rats.

### SPS-induced changes in anxiety-related behavior in the EPM

**Fig. 2:**
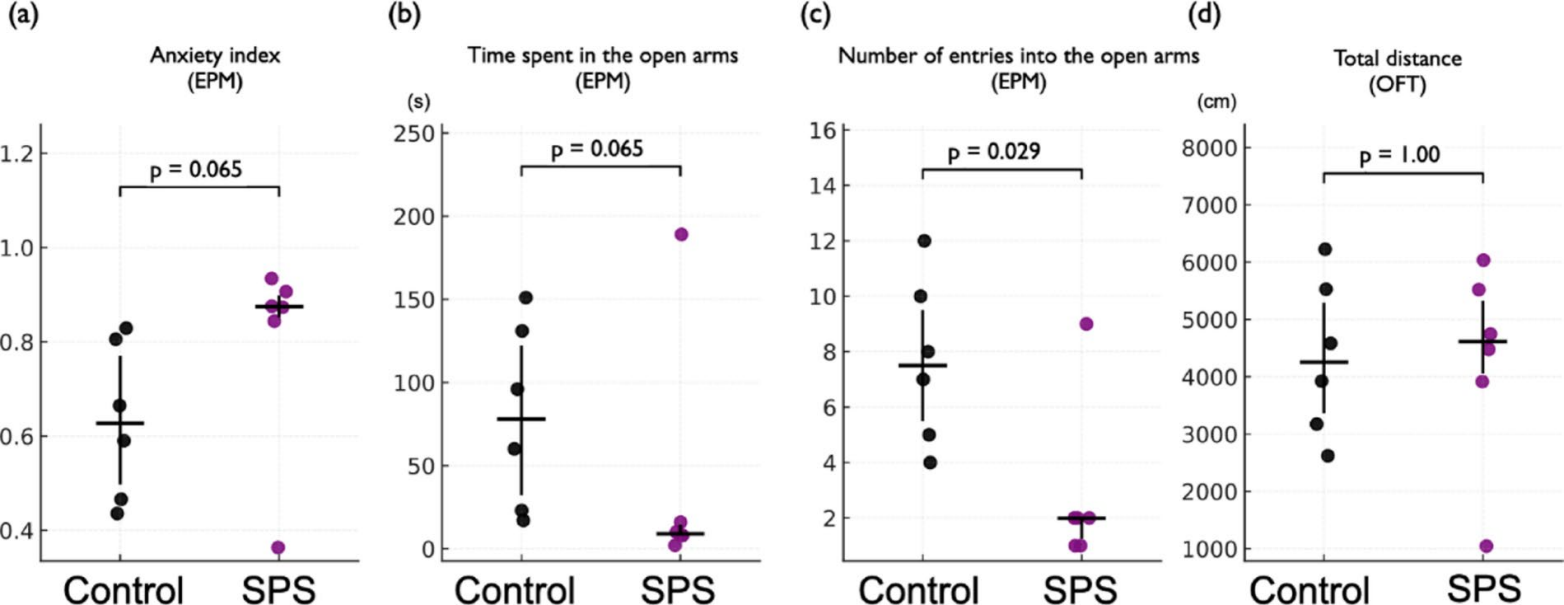
SPS-induced changes in anxiety-related behavior in the EPM. Rats were exposed to the SPS paradigm, after which anxiety-related behavior was evaluated using the EPM, and locomotor activity was assessed using the OFT. (**a**) Anxiety index: SPS rats showed a higher anxiety index than controls (median [IQR]; Control: 0.63 [0.50–0.77], SPS: 0.87 [0.85–0.90], *p* = 0.065). (**b**) Time spent in open arms: SPS rats spent less time in the open arms of the EPM than controls (Control: 78.00 [32.25–122.25], SPS: 9.00 [8.00–14.50], *p* = 0.065). (**c**) Number of open-arm entries: SPS rats made significantly fewer open-arm entries than controls (Control: 7.50 [5.50–9.50], SPS: 2.00 [1.25–2.00], *p* = 0.029). (**d**) Locomotor activity: No significant difference was observed between groups in total distance traveled in the OFT (Control: 4,254.81 [3,361.84–5,292.37], SPS: 4,612.97 [4,055.98–5,327.68], *p* = 1.00). These results indicate that SPS exposure is associated with anxiety-like behavior changes without affecting locomotor activity. Data are shown as scatter plots indicating the median and IQR. The Mann–Whitney U test was used for statistical comparisons. Time variables are expressed in seconds, and distance is expressed in centimeters. Sample size: *n* = 6 per group. EPM, elevated plus maze; OFT, open field test; SPS, single prolonged stress; IQR, interquartile range.

First, we assessed the behavioral effects of SPS exposure in an independent cohort (Experiment 1) by examining anxiety-related behaviors in the EPM. Consistent with our previous findings^20^, SPS-exposed rats exhibited increased anxiety-like behavior compared to control rats. Although the anxiety index (*p* = 0.065) (Fig. 2a) and time spent in the open arms (*p* = 0.065) (Fig. 2b) did not reach statistical significance, both measures showed a trend toward increased anxiety-like behavior. The number of open-arm entries was significantly lower in the SPS group (*p* = 0.029) (Fig. 2c). These findings are consistent with those of previous studies and suggest that SPS exposure leads to heightened anxiety-related behaviors in the EPM. Locomotor activity, assessed by the total distance traveled in the open field test (OFT), did not differ significantly between the groups (*p* = 1.00) (Fig. 2d). The time spent in the center zone of the OFT did not differ between groups (*p* = 0.80) (Supplementary Fig. 1). All groups consisted of 6 rats.

### Oral MCT oil treatment attenuated anxiety-related behavior in SPS-exposed rats

**Fig. 3:**
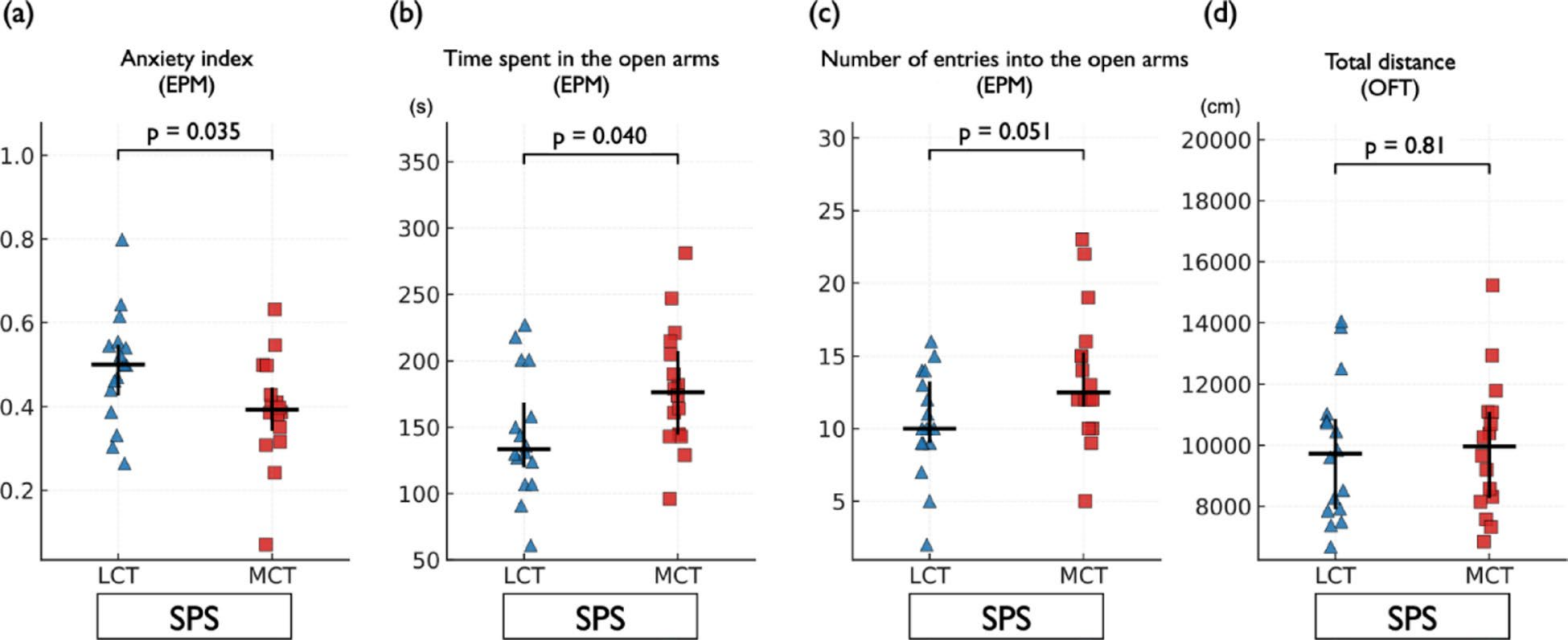
Oral MCT oil treatment attenuated anxiety-like behavior in SPS-exposed rats. Rats were subjected to the SPS procedure and subsequently administered 0.8 mL of either LCT oil or MCT oil daily for 2 weeks. Anxiety-like behavior was evaluated using the EPM, and locomotor activity and anxiety-related behavior were assessed using the OFT. (**a**) Anxiety index: The MCT group showed a significantly lower anxiety index compared with the LCT group (median [IQR]; LCT: 0.50 [0.43–0.55], MCT: 0.39 [0.34–0.45], *p* = 0.035). (**b**) Time spent in the open arms: MCT-treated rats spent significantly more time in the open arms of the EPM than LCT-treated controls (LCT: 133.50 [119.75–168.75], MCT: 176.50 [144.50–207.50], *p* = 0.040). (**c**) Number of open-arm entries: MCT treatment tended to increase open-arm entries (LCT: 10.00 [9.00–13.25], MCT: 12.50 [11.50–15.25], *p* = 0.051). (**d**) Locomotor activity: No significant difference was observed between groups in total distance traveled in the OFT (LCT: 9,724.60 [7,904.94–10,866.50], MCT: 9,959.53 [8,270.52–11,085.61], *p* = 0.81). These results indicate that oral MCT oil administration affected anxiety-like behavior in SPS-exposed rats without affecting locomotor activity. Data are shown as scatter plots indicating the median and IQR. The Mann–Whitney U test was used for statistical comparisons. Time variables are expressed in seconds, and distance is expressed in centimeters. Sample size: *n* = 16 per group. EPM, elevated plus maze; OFT, open field test; MCT, medium-chain triglyceride; LCT, long-chain triglyceride; SPS, single prolonged stress; IQR, interquartile range.

Next, in a separate cohort tested in an independent experimental batch (Experiment 2), we investigated whether repeated oral administration of MCT oil modulated anxiety-like behavior in SPS-exposed rats. In the EPM, MCT-treated rats showed a significant reduction in the anxiety index compared to the LCT group (*p* = 0.035) (Fig. 3a). MCT oil significantly increased the time spent in the open arms (*p* = 0.040) (Fig. 3b) and tended to increase the number of open-arm entries (*p* = 0.051) (Fig. 3c). Locomotor activity in the OFT was not significantly altered (*p* = 0.81) (Fig. 3d). The time spent in the center zone of the OFT did not differ (*p* = 0.28) (Supplementary Fig. 1). All groups consisted of 16 rats.

### MCT alone did not alter anxiety-like behavior, locomotor activity, or body weight

To determine whether MCT oil alone affects anxiety-related behavior, we assessed rats after 2 weeks of oral MCT administration without exposure to the SPS paradigm in a separate cohort (Experiment 3). MCT treatment did not alter the anxiety index (*p* = 0.29), time spent in the open arms (*p* = 0.69), or the number of open-arm entries (*p* = 0.73) in the EPM. Furthermore, locomotor activity in the OFT did not differ between groups (*p* = 0.78). The time spent in the center zone of the OFT did not differ (*p* = 0.54) (Supplementary Fig. 1). Detailed behavioral data are presented in Supplementary Fig. 2. To assess the safety of repeated oral MCT administration, body weight was monitored during the experimental period in stress-naïve rats. Body weight increased over time in both groups, and no significant differences were observed between the MCT- and LCT-treated rats at either 1 week (*p* = 0.135) or 2 weeks (*p* = 0.196) (Supplementary Fig. 3). All groups consisted of 16 rats.

### Effects of MCT oil on baseline serum BDNF and cytokine levels in SPS rats

To evaluate the baseline molecular effects of oral MCT oil administration in SPS-exposed rats, BDNF and cytokine levels were measured without additional acute stress. The following three groups were compared: non-SPS + LCT, SPS + LCT, and SPS + MCT. After 2 weeks of daily MCT or LCT oil administration, BDNF, IL-1β, interleukin-6 (IL-6), and TNF-α were quantified in the serum (protein levels). No significant group differences were found in serum BDNF and cytokine levels (Supplementary Fig. 4). All groups consisted of 7 rats.

### MCT oil was associated with differences in BDNF responses to acute restraint stress in SPS rats

**Fig. 4:**
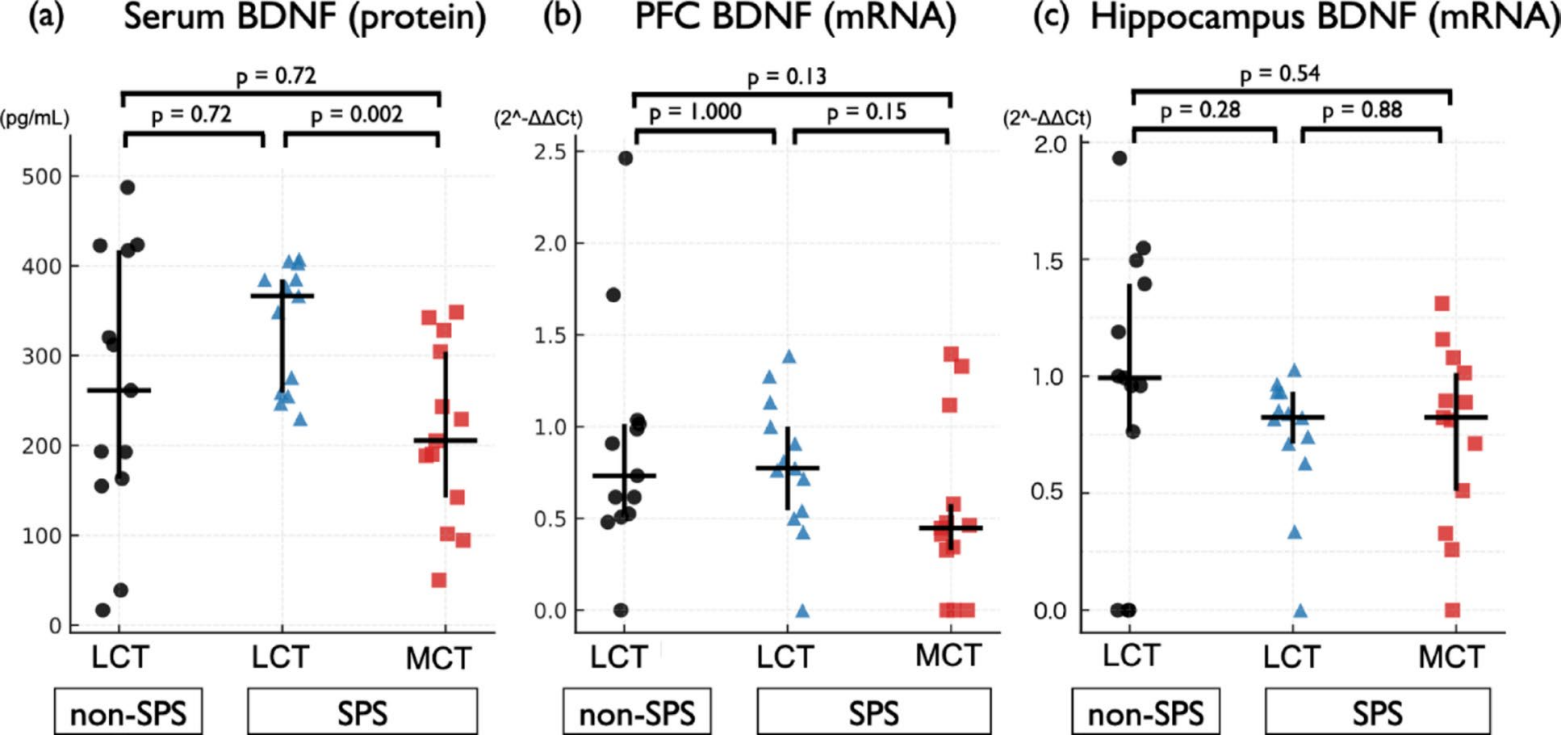
MCT oil was associated with differences in BDNF responses to acute restraint stress in SPS rats. Rats were subjected to 1 h of acute restraint stress immediately before euthanasia, and serum and brain samples were collected for BDNF analysis. (**a**) Serum BDNF levels (median [IQR]; non-SPS + LCT: 261.3 [163.1–417.1], SPS + LCT: 366.6 [258.9–385.0], SPS + MCT: 205.4 [142.3–304.4]). Pairwise comparisons: non-SPS + LCT vs. SPS + LCT, *p* = 0.72; non-SPS + LCT vs. SPS + MCT, *p* = 0.72; SPS + LCT vs. SPS + MCT, *p* = 0.002. (**b**) BDNF mRNA expression in the PFC (median [IQR]; non-SPS + LCT: 0.732 [0.525–1.014], SPS + LCT: 0.774 [0.543–1.000], SPS + MCT: 0.448 [0.328–0.578]). Pairwise comparisons: non-SPS + LCT vs. SPS + LCT, *p* = 1.000; non-SPS + LCT vs. SPS + MCT, *p* = 0.13; SPS + LCT vs. SPS + MCT, *p* = 0.15. (**c**) BDNF mRNA expression in the hippocampus (median [IQR]; non-SPS + LCT: 0.993 [0.763–1.395]; SPS + LCT: 0.824 [0.712–0.933]; SPS + MCT: 0.824 [0.511–1.014]). Pairwise comparisons: non-SPS + LCT vs. SPS + LCT, *p* = 0.28; non-SPS + LCT vs. SPS + MCT, *p* = 0.54; SPS + LCT vs. SPS + MCT, *p* = 0.88. These findings indicate that serum BDNF levels under acute restraint stress were significantly lower in the MCT-treated group than in the LCT-treated group. No significant differences were detected in BDNF mRNA expression in the PFC or hippocampus; however, a non-significant trend toward lower expression was observed in the MCT-treated group in the PFC. Data are presented as scatter plots showing the median and IQR. Pairwise Mann–Whitney U tests were performed for group comparisons, and p-values were adjusted using the Holm method. Serum BDNF concentrations are expressed in pg/mL as measured by ELISA, whereas PFC and hippocampal BDNF values represent relative mRNA expression levels calculated using the 2^-ΔΔCt method. Sample size: *n* = 13 rats per group. BDNF, brain-derived neurotrophic factor; PFC, prefrontal cortex; SPS, single prolonged stress; LCT, long-chain triglyceride; MCT, medium-chain triglyceride; IQR, interquartile range.

To investigate molecular responses to acute restraint stress in SPS-exposed rats and the potential modulatory effects of MCT oil, we conducted a second experiment in which animals were subjected to a 1-h restraint stress immediately before euthanasia. Under these conditions, serum BDNF levels were significantly lower in the SPS + MCT group than in the SPS + LCT group (*p* = 0.002). No significant differences were observed between the non-SPS + LCT group and either the SPS + LCT or SPS + MCT groups (*p* = 0.72 for both) (Fig. 4a). In the PFC, BDNF mRNA expression did not differ significantly among groups; however, the SPS + MCT group showed a non-significant trend toward lower expression compared with the SPS + LCT (*p* = 0.15) and non-SPS + LCT (*p* = 0.13) groups. No difference was observed between the non-SPS + LCT and SPS + LCT groups (*p* = 1.00) (Fig. 4b). In the hippocampus, BDNF mRNA expression did not differ significantly among groups. No significant group differences were detected in IL-1β, IL-6, or TNF-α expression in the serum or across any brain regions under these acutely stressed conditions (Supplementary Fig. 5). All groups consisted of 13 rats.

## Discussion

This study examined the effects of orally administered MCT oil on behavioral and molecular stress responses in rats subjected to the SPS paradigm, a stress model widely used in PTSD research. Daily MCT supplementation increased circulating BHB levels and was associated with reduced anxiety-like behavior. In addition, repeated MCT administration did not affect body weight compared with LCT-treated controls. At the molecular level, MCT treatment was accompanied by lower serum BDNF levels under conditions of acute restraint stress in SPS-exposed rats compared with the LCT-treated group. In the PFC, BDNF mRNA expression did not show statistically significant group differences, although a similar directional trend to that observed in serum BDNF was noted. These behavioral effects occurred in parallel with differences in serum BDNF responses during acute stress, suggesting a potential association between metabolic changes and stress-related molecular responses. However, it should be noted that the behavioral assessments in the present study were limited to anxiety-related behavior under SPS conditions and did not capture the full spectrum of PTSD-related phenotypes.

Recently, ketone bodies have attracted increasing attention as potential therapeutic agents for mood and anxiety-related disorders. Animal and human studies have suggested beneficial behavioral effects of exogenous ketone supplementation. We previously reported that BHB administration ameliorated behavioral abnormalities in animal models of depression^18,19^ and PTSD^20^, and that MCT administration improved depressive-like behavior in depression models^28^. We also hypothesized that inhibition of NLRP3 inflammasome activation by BHB contributes to the prevention of stress-related disorders^29^. Preclinical experiments have shown that exogenous ketone supplementation reduces anxiety-like behavior in rodents^30^, whereas a clinical pilot study demonstrated that a ketogenic diet supplemented with BHB salts produced clinically meaningful improvements in PTSD symptoms and quality of life^31^. Although preliminary, these findings suggest that BHB-based metabolic interventions may be feasible and potentially effective under stress-related conditions. In the present study, repeated MCT administration did not affect body weight, supporting its short-term tolerability under our experimental conditions. However, the mechanisms underlying its behavioral effects remain unclear, and neurotrophic modulation—particularly through BDNF—has been proposed as a possible pathway.

Supporting this hypothesis, numerous studies have linked PTSD to alterations in BDNF signaling. In clinical populations, patients with chronic PTSD often exhibit reduced serum or plasma BDNF levels, suggesting that long-term suppression of BDNF may serve as a peripheral marker of disease chronicity^32^. In contrast to these chronic changes, BDNF levels can acutely increase in response to stress exposure. In healthy humans, the Trier Social Stress Test induces a transient rise in serum BDNF^33^, and similar effects have been observed in rodents following acute immobilization or restraint stress^34,35^. Clinical evidence also supports this biphasic pattern: shortly after trauma, serum BDNF levels are often elevated, as reported in patients with acute stress disorder or newly diagnosed PTSD, before declining with symptom improvement^36^. Supporting this, individuals evaluated within 1 year after trauma exposure exhibited significantly higher serum BDNF levels than healthy controls, whereas this effect was absent in those evaluated more remotely from the trauma^37^. Moreover, elevated BDNF levels measured immediately after a motor vehicle accident have been associated with an increased risk of subsequent PTSD symptom development^38^. These observations suggest that transient BDNF elevation following acute stress may contribute to the formation or consolidation of trauma-related memories, preceding the decline observed in chronic PTSD.

In the present study, we did not observe a clear increase in serum BDNF following acute restraint stress in SPS rats. However, group differences were observed under stress conditions, with MCT-treated animals showing lower serum BDNF levels during acute restraint stress. These findings raise the possibility that MCT administration may alter BDNF responses during stress re-exposure, although the functional significance of such changes and their relationship to behavioral outcomes remain unclear. In this context, animal studies have suggested that reactivation of stress-related memories in PTSD models is associated with changes in BDNF expression. In particular, region-specific increases in BDNF have been reported in the medial prefrontal cortex and amygdala, indicating dynamic synaptic plasticity within fear-related circuits during memory retrieval following secondary stress exposure^39^. Importantly, both that study and the present work focused on molecular responses elicited by additional stressors applied after PTSD-like pathology had been induced, rather than on baseline alterations, highlighting a shared emphasis on BDNF responses during re-exposure to stress. In the present study, MCT administration was associated with lower serum BDNF levels following acute restraint stress, whereas BDNF mRNA expression in the PFC did not differ significantly among groups. Accordingly, these findings should be interpreted with caution; however, they raise the possibility that changes in BDNF responses during re-exposure to stress may be involved in PTSD-related pathophysiology.

This study has several limitations. First, the behavioral assessment in the present study focused primarily on anxiety-like behavior evaluated using the EPM. PTSD encompasses a broad range of symptoms, including hyperarousal and exaggerated startle responses, which were not assessed in the current experimental design. Paradigms such as foot shock–induced fear conditioning and fear-potentiated startle are widely used to model these features of PTSD and have been pharmacologically validated for translational relevance^40^. Incorporating such behavioral assays in future studies would enable a more comprehensive evaluation of PTSD-like phenotypes and provide deeper insight into the therapeutic potential of MCT or BHB interventions. Therefore, caution is warranted when interpreting the present findings in relation to the broader pathophysiology of PTSD. Second, the behavioral experiments were conducted in separate experimental batches at different time points using independent cohorts. The magnitude of the SPS effect appeared to differ across experiments, which may reflect variability between experimental batches. Because stress condition was confounded with batch, the datasets were not pooled for a factorial two-way ANOVA. Accordingly, the results were interpreted within the context of each experiment rather than directly compared across experiments. Future studies in which all four groups are generated and tested concurrently will be required to enable formal factorial inference. Third, only male rats were used, and potential sex differences were not examined. The prevalence, severity, and burden of PTSD are generally higher in women than in men^41^. Future studies incorporating female animals are needed to explore possible sex-dependent effects. Fourth, BDNF expression was assessed at only two time points—2 weeks after SPS and 1 h after acute restraint stress. This limited sampling precludes a comprehensive understanding of the temporal BDNF dynamics. Clinical studies have frequently reported reduced BDNF levels in individuals with chronic PTSD, suggesting a biphasic trajectory characterized by a transient increase following acute stress and a subsequent decline with prolonged exposure. Therefore, future research should include additional time points to clarify these temporal patterns. Fifth, molecular analyses were confined to the serum, PFC, and hippocampus. The amygdala—a key region involved in fear memory and emotion regulation—was not examined. Including this and other relevant regions in future studies may help elucidate region-specific patterns of neuroplasticity. Sixth, BDNF was assessed as total BDNF in the present study, without distinguishing between its functionally distinct isoforms, such as mature BDNF (mBDNF) and proBDNF. Because these isoforms are known to exert different effects on synaptic plasticity through distinct receptor systems^42^, it remains unclear which component of BDNF signaling contributes to the observed behavioral and molecular effects. Future studies employing isoform-specific analyses will be necessary to clarify the relative roles of mBDNF- and proBDNF-dependent signaling pathways in stress-induced neuroplastic changes. Seventh, although BHB is known to act through multiple molecular pathways—including inhibition of histone deacetylases (HDACs), activation of hydroxycarboxylic acid receptor 2 (HCAR2)^43,44^, and modulation of mitochondrial function^45^ and cellular energy metabolism^22^—these mechanisms were not directly assessed in the present study. In addition, MCT may influence brain function through BHB-independent mechanisms such as alterations in gut microbiota^46^. Therefore, the precise pathways through which MCT administration influenced behavioral and molecular responses in this study remain unclear.

In summary, oral MCT oil administration attenuated anxiety-like behavior under SPS conditions and was associated with differences in serum BDNF responses under acute stress. These findings suggest that MCT-derived metabolic changes may influence stress-related behavioral responses. Further studies are required to clarify the underlying mechanisms and the potential translational relevance of these findings for stress-related disorders.

## Methods

### Animals and housing

Male wild-type Sprague-Dawley (SD) rats (6 weeks old, weighing 200–220 g), with no prior exposure to drugs or experimental procedures, were obtained from Charles River Laboratories (Yokohama, Japan). Rats were group-housed, three per cage, under controlled environmental conditions: a 12-h light/dark cycle (lights on at 7:30 AM and off at 7:30 PM), constant temperature (25 °C), and ad libitum access to standard rodent chow (Rodent Diet CE-2, CLEA Japan, Tokyo, Japan) and tap water. Prior to the initiation of experimental procedures, rats were acclimated to housing conditions for 7 days. After the experiments, rats were euthanized by decapitation without drug administration. All procedures were conducted in accordance with the ethical guidelines of the Tottori University Animal Care and Use Committee (IRB approval number: h34-Y004) and complied with institutional regulations. Every effort was made to reduce the number of animals used and to minimize pain, discomfort, and distress. The study is reported in accordance with ARRIVE guidelines.

### MCT and LCT oil administration

Nisshin MCT Oil^®^ (Nisshin Oillio, Tokyo, Japan) was used as the MCT source. To match calorie content and dosage between groups, Nisshin Salad Oil^®^ (Nisshin Oillio, Tokyo, Japan) was used as the LCT source. MCT or LCT oil was administered intragastrically at a volume of 0.8 mL per rat once daily in the morning, starting the day after SPS exposure and continuing until the day of euthanasia.

### Measurement of blood BHB concentration

To assess the temporal profile of blood BHB concentration following oral oil administration, rats were divided into two groups receiving either MCT oil or LCT oil. Each rat received 0.8 mL of the assigned oil via intragastric gavage (MCT group, *n* = 5; LCT group, *n* = 4). Approximately 10 µL of blood was collected from the tail vein of the same rats at the following time points: prior to oil administration (baseline) and at 15 min, 30 min, 1 h, 3 h, and 6 h post-administration. Blood BHB levels were measured using a handheld glucose/BHB meter (FreeStyle Precision Neo; Abbott, Japan)^20^.

### SPS procedure

The SPS protocol was used to induce PTSD-like phenotypes in rats. On the day of the procedure, rats were first subjected to 2 h of immobilization stress, followed immediately by a 20-min forced swim in a plastic container (32 cm diameter × 47 cm height) filled with water to a depth of 34 cm. After swimming, the rats were allowed a 15-min recovery period before exposure to diethyl ether to achieve light anesthesia, as previously described^47,48^. Following exposure to the stressors, rats were group-housed (three per cage) and left undisturbed except for routine oil administration and behavioral testing.

### Experimental design

To examine the effects of MCT oil in an animal model of PTSD, two sets of experiments were conducted: (1) behavioral experiments and (2) assessment of serum protein and brain mRNA expression levels. Rats were randomly assigned to each experimental group without consideration of baseline characteristics. All experimental procedures were standardized within each experiment to minimize confounders.

### (1) Behavioral experiments

Three independent behavioral experiments were conducted to evaluate the effects of MCT oil on anxiety-like behavior in the context of PTSD.

Experiment 1: Validation of the SPS procedure as a PTSD model. Rats were divided into two groups: unstressed controls and SPS-exposed rats (*n* = 6 per group). Behavioral testing was performed to confirm the emergence of anxiety-like behaviors following SPS exposure.

Experiment 2: Evaluation of anxiety-like behavior in SPS-exposed rats following MCT administration. On day 1, rats underwent SPS. From days 2 to 15, they received a daily dose of 0.8 mL of either MCT or LCT oil (control) via intragastric gavage in the morning (*n* = 16 per group). On day 8, rats were habituated to the EPM as previously described^20^. The OFT was conducted on day 14, followed by the EPM test on day 15.

**Fig. 5:**
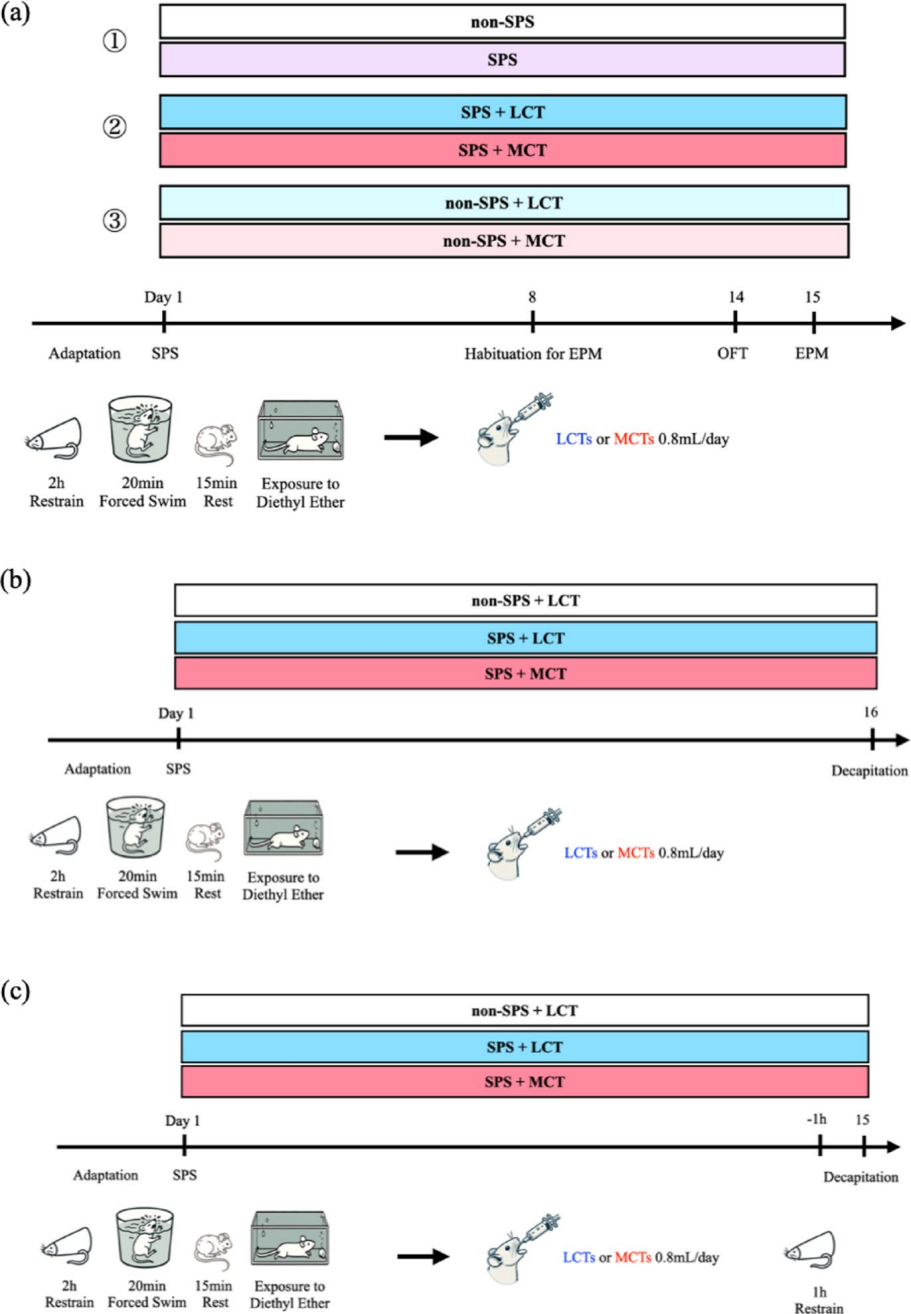
Experimental design and timeline of behavioral and molecular analyses. (**a**) Behavioral experiments. Three independent behavioral experiments were conducted to evaluate the effects of MCT oil on anxiety-like behaviors in PTSD. In Experiment 1, rats were divided into non-stressed (non-SPS) and SPS-exposed groups (*n* = 6 each) to validate the SPS model. Experiment 2 examined whether MCT oil reduced anxiety-like behavior in SPS-exposed rats. On day 1, rats underwent SPS, followed by daily oral administration of 0.8 mL MCT or LCT oil via intragastric gavage from days 2 to 15 (*n* = 16 each). On day 8, rats were habituated to the EPM, followed by the OFT on day 14 and the EPM test on day 15 to assess anxiety-like behavior. Experiment 3 investigated whether MCT oil influenced baseline anxiety levels in stress-naïve rats, which received MCT or LCT oil under the same dosing schedule (*n* = 16 each) without SPS exposure. All behavioral tests were performed during the light phase. (**b**) Molecular analysis under non-stress conditions. This experiment examined the effects of MCT oil on baseline BDNF and cytokine expression in SPS rats. Rats were assigned to three groups: non-SPS + LCT, SPS + LCT, and SPS + MCT (*n* = 7 each). SPS was performed on day 1, followed by daily oral administration of 0.8 mL MCT or LCT oil. On day 16, rats were euthanized, and serum samples were collected for BDNF and cytokine analyses (ELISA). (**c**) Molecular analysis after acute restraint stress. This experiment evaluated whether MCT oil modulates molecular responses to acute stress in SPS rats. Rats were assigned to three groups: non-SPS + LCT, SPS + LCT, and SPS + MCT (*n* = 13 each). After 2 weeks of oil administration, rats were subjected to 1-h restraint stress immediately before decapitation on day 15. Serum, PFC, and hippocampal samples were collected to measure BDNF and cytokine protein and mRNA levels. SPS, single prolonged stress; EPM, elevated plus maze; OFT, open field test; LCT, long-chain triglyceride; MCT, medium-chain triglyceride; BDNF, brain-derived neurotrophic factor; PFC, prefrontal cortex.

Experiment 3: Evaluation of MCT effects in stress-naïve rats. Rats were divided into MCT-treated and LCT-treated groups (*n* = 16 per group) without exposure to SPS. From days 1 to 15, rats received a daily dose of 0.8 mL of either MCT or LCT oil via intragastric gavage in the morning. Behavioral testing followed the same schedule as in Experiment 2. This experiment determined whether MCT oil alone could alter baseline anxiety levels without SPS exposure. Body weight was measured at baseline and weekly during the experimental period to monitor potential effects of repeated oil administration on general physiological status. All behavioral tests were performed during the light phase in a dedicated testing room. The experimental design is illustrated in Fig. 5a. Because the behavioral experiments (Experiments 1–3) were conducted as independent experimental batches at different time points using separate cohorts, the datasets were not pooled for a factorial analysis across stress conditions, and the results were interpreted within each experiment rather than directly compared across experiments.

### OFT

The OFT was performed to evaluate locomotor activity and anxiety-like behavior. Rats were individually placed in a square open-field arena (90 × 90 × 45 cm; LE800S-S, Panlab, USA) and allowed to explore freely for 10 min. The center zone was defined as the central 50% of the total arena area^49^. The arena was thoroughly cleaned between trials to eliminate scent cues, and all sessions were conducted under standard lighting conditions^20^. Animal movements were recorded using a video-tracking system (SMART 3.0, Panlab, USA), and the total distance traveled and the time spent in the center zone were measured.

### EPM Test

The EPM (LE840A-S, Panlab, USA) was used to assess anxiety-like behavior. The apparatus consisted of two open arms (50 × 10 cm) and two closed arms (50 × 10 cm, enclosed by 30 cm-high walls), arranged in a plus shape and elevated 50 cm above the floor. On day 8, rats were habituated to the maze for 5 min to reduce the risk of falling, and the formal test was conducted on day 15. Both sessions were conducted in the dark to minimize external stress. In each session, rats were placed at the end of a closed arm facing the center and allowed to explore the maze freely for 5 min. All trials were recorded and analyzed by an observer blinded to the group allocation. The apparatus was cleaned thoroughly after each trial^20^. Arm entry was defined as a rat entering an arm with more than half of its body. The following parameters were recorded: time spent in open and closed arms, number of entries into each arm type, and total arm entries. To quantify anxiety-like behavior, an anxiety index^50^, was calculated as follows:

$${\text{Anxiety index}} = 1-({\frac{\text{Time in open arms}}  {\text{Total time on maze}}}+\frac{{\text{Number of open}} - {\text{arms entries}}} {\text{Total arm entries}})/2$$

The index ranges from 0 to 1, with higher values indicating greater anxiety-like behavior.

### (2) Assessment of serum protein levels and mRNA expression in the brain

Two independent experiments were conducted to investigate the effects of MCT oil on molecular responses in the PTSD model. Behavioral testing was not performed in either experiment to eliminate potential confounding effects of testing-related stress. In both experiments, rats were assigned to three groups: non-SPS + LCT, SPS + LCT, and SPS + MCT (*n* = 7 per group in the first experiment; *n* = 13 per group in the second experiment). On day 1, rats in the SPS groups were subjected to the SPS procedure. From day 2 onward, all rats received a daily dose of 0.8 mL of either MCT or LCT oil via intragastric gavage.

The first experiment aimed to evaluate baseline expression levels of serum BDNF and cytokines in PTSD model rats and to determine the modulatory effects of MCT oil on these molecular markers. On day 16, rats were euthanized, and blood samples were collected. Serum BDNF and cytokine protein levels were analyzed (Fig. 5b).

The second experiment aimed to evaluate molecular responses to acute restraint stress in a PTSD rat model and to determine whether oral MCT oil modulates BDNF and cytokine responses to stress. On day 15, rats were subjected to a 1-h restraint stress immediately before decapitation, after which they were euthanized and blood samples were collected. Serum BDNF and cytokine protein levels, as well as mRNA expression of BDNF and cytokines in the PFC and hippocampus, were analyzed (Fig. 5c).

### Serum BDNF and cytokine measurement

Blood samples were collected and stored at 4 °C overnight. After centrifugation, the supernatant was collected and stored at − 80 °C until analysis. Serum levels of BDNF, TNF-α, IL-1β and IL-6 were measured using commercially available ELISA kits according to the manufacturer’s instructions (BDNF: ab213899; TNF-α: ab236712; IL-1β: ab255730; IL-6: ab100772; all from Abcam, Cambridge, United Kingdom).

### mRNA expression analysis in the brain

Total RNA was extracted from the PFC and hippocampus. After homogenization using the Multi-beads Shocker (Yasui Kikai Corporation, Osaka, Japan), RNA was purified with the RNeasy Plus Universal Mini Kit (QIAGEN, Venlo, Netherlands) following the manufacturer’s protocol. First-strand cDNA synthesis was performed using 2 µg of RNA according to standard methods. Quantitative real-time PCR was conducted using the ViiA 7 Real-Time PCR System (Thermo Fisher Scientific, Waltham, MA, USA) along with TaqMan Gene Expression Assays (Thermo Fisher Scientific) to evaluate the expression levels of TNF-α, IL-1β, IL-6, BDNF, and β-actin (ACTB) mRNA. After confirming with a small number of samples that there were no issues with the amplification efficiency of each gene, each target gene was measured in duplicate, and the mean threshold cycle (Ct) values were used for analysis. Relative gene expression was calculated using the comparative threshold cycle method (ΔΔCt), with ACTB as the internal control. Ct values ≥ 36 were considered below the detection threshold and were included in the statistical analysis.

### Statistical analyses

All statistical analyses were performed using EZR (Saitama Medical Center, Jichi Medical University, Japan), a modified version of R Commander that includes additional functions commonly used in biostatistics^51^. Sample sizes for behavioral and molecular experiments were determined based on data from our previous study investigating the effects of BHB^20^. To confirm the increase in blood BHB levels following MCT oil administration, Mann–Whitney U tests were performed. The Mann–Whitney U test was also used for comparisons between two groups (e.g., behavioral tests). For comparisons among three groups (e.g., serum cytokine measurements), pairwise Mann–Whitney U tests were performed, and p-values were adjusted using the Holm method to correct for multiple comparisons.

Data are presented as scatter plots with medians and interquartile ranges. Statistical significance was set at *p* < 0.05.

### 3Rs in this study

This study used rats to investigate the in vivo effects of MCT oil on PTSD-related pathophysiology and stress responses. The minimum number of animals required to achieve statistical validity was estimated before the start of the study, and all efforts were made to avoid unnecessary animal use. Although procedures such as SPS, oral gavage, and behavioral testing may have caused transient physical or psychological stress, every effort was made to minimize pain, suffering, and distress. Animals were monitored daily for health and well-being, and all experimental protocols were conducted in accordance with institutional and national guidelines for animal care and use.

## Supplementary Information

Below is the link to the electronic supplementary material.

Supplementary Material 1

## Data Availability

The data presented in the main figures and tables supporting the findings of this study are available from the corresponding author upon reasonable request.

## References

[1] Bisson, J. I., Cosgrove, S., Lewis, C. & Roberts, N. P. Post-traumatic stress disorder. *BMJ***h6161**10.1136/bmj.h6161 (2015). 10.1136/bmj.h6161 PMC4663500 26611143

[2] Gu, W., Wang, C., Li, Z., Wang, Z. & Zhang, X. Pharmacotherapies for Posttraumatic Stress Disorder: A Meta-Analysis. *J. Nerv. Mental Disease*. **204**, 331–338. 10.1097/NMD.0000000000000478 (2016). 10.1097/NMD.0000000000000478 26894318

[3] Yu, Y. H. et al. The Medial Prefrontal Cortex, Nucleus Accumbens, Basolateral Amygdala, and Hippocampus Regulate the Amelioration of Environmental Enrichment and Cue in Fear Behavior in the Animal Model of PTSD. *Behav. Neurol.* **2022**, 1–14. 10.1155/2022/7331714 (2022). 10.1155/2022/7331714 PMC8843982 35178125

[4] Yang, J. J. & Jiang, W. Immune biomarkers alterations in post-traumatic stress disorder: A systematic review and meta-analysis. *J. Affect. Disord.* **268**, 39–46. 10.1016/j.jad.2020.02.044 (2020). 32158005 10.1016/j.jad.2020.02.044

[5] Hussein, S., Dalton, B., Willmund, G. D., Ibrahim, M. A. A. & Himmerich, H. A systematic review of tumor necrosis factor-α in post-traumatic stress disorder: evidence from human and animal studies. *Psychiatr Danub*. **29**, 407–420. 10.24869/psyd.2017.407 (2017). 29197197 10.24869/psyd.2017.407

[6] Hori, H. & Kim, Y. Inflammation and post-traumatic stress disorder. *Psychiatry Clin. Neurosci.* **73**, 143–153. 10.1111/pcn.12820 (2019). 30653780 10.1111/pcn.12820

[7] Andero, R. & Ressler, K. J. Fear extinction and BDNF: translating animal models of PTSD to the clinic. *Genes Brain Behav.* **11**, 503–512. 10.1111/j.1601-183X.2012.00801.x (2012). 22530815 10.1111/j.1601-183X.2012.00801.x PMC3389160

[8] Lee, B. et al. Effects of systemic administration of ibuprofen on stress response in a rat model of post-traumatic stress disorder. *Korean J. Physiol. Pharmacol.* **20**, 357. 10.4196/kjpp.2016.20.4.357 (2016). 27382352 10.4196/kjpp.2016.20.4.357 PMC4930904

[9] Lee, B., Shim, I., Lee, H. & Hahm, D. H. Effect of oleuropein on cognitive deficits and changes in hippocampal brain-derived neurotrophic factor and cytokine expression in a rat model of post-traumatic stress disorder. *J. Nat. Med.* **72**, 44–56. 10.1007/s11418-017-1103-8 (2018). 28884427 10.1007/s11418-017-1103-8

[10] Zhao, M. et al. Long-Term Effect of Post-traumatic Stress in Adolescence on Dendrite Development and H3K9me2/BDNF Expression in Male Rat Hippocampus and Prefrontal Cortex. *Front. Cell. Dev. Biol.* **8**, 682. 10.3389/fcell.2020.00682 (2020). 32850808 10.3389/fcell.2020.00682 PMC7412801

[11] Zhao, M. et al. Effects of traumatic stress in adolescence on PTSD-like behaviors, dendrite development, and H3K9me2/BDNF expression in the amygdala of male rats. *J. Affect. Disord.* **296**, 388–399. 10.1016/j.jad.2021.09.101 (2022). 34619155 10.1016/j.jad.2021.09.101

[12] Dell’Osso, L. et al. Brain-derived neurotrophic factor plasma levels in patients suffering from post-traumatic stress disorder. *Prog. Neuropsychopharmacol. Biol. Psychiatry*. **33**, 899–902. 10.1016/j.pnpbp.2009.04.018 (2009). 19409951 10.1016/j.pnpbp.2009.04.018

[13] Newman, J. C. & Verdin, E. Ketone bodies as signaling metabolites. *Trends Endocrinol. Metabolism*. **25**, 42–52. 10.1016/j.tem.2013.09.002 (2014). 10.1016/j.tem.2013.09.002 PMC4176946 24140022

[14] Cotter, D. G., Schugar, R. C. & Crawford, P. A. Ketone body metabolism and cardiovascular disease. *Am. J. Physiol. Heart Circ. Physiol.* **304**, H1060–H1076. 10.1152/ajpheart.00646.2012 (2013). 23396451 10.1152/ajpheart.00646.2012 PMC3625904

[15] Youm, Y. H. et al. The ketone metabolite β-hydroxybutyrate blocks NLRP3 inflammasome–mediated inflammatory disease. *Nat. Med.* **21**, 263–269. 10.1038/nm.3804 (2015). 25686106 10.1038/nm.3804 PMC4352123

[16] Marosi, K. et al. 3-Hydroxybutyrate regulates energy metabolism and induces BDNF expression in cerebral cortical neurons. *J. Neurochem.* **139**, 769–781. 10.1111/jnc.13868 (2016). 27739595 10.1111/jnc.13868 PMC5123937

[17] Sleiman, S. F. et al. Exercise promotes the expression of brain derived neurotrophic factor (BDNF) through the action of the ketone body β-hydroxybutyrate. *eLife* 5, e15092 (2016). 10.7554/eLife.15092 10.7554/eLife.15092 PMC4915811 27253067

[18] Yamanashi, T. et al. Beta-hydroxybutyrate, an endogenic NLRP3 inflammasome inhibitor, attenuates stress-induced behavioral and inflammatory responses. *Sci. Rep.* **7**, 7677. 10.1038/s41598-017-08055-1 (2017). 28794421 10.1038/s41598-017-08055-1 PMC5550422

[19] Kajitani, N. et al. Prefrontal cortex infusion of beta-hydroxybutyrate, an endogenous NLRP3 inflammasome inhibitor, produces antidepressant‐like effects in a rodent model of depression. *Neuropsychopharm Rep.* **40**, 157–165. 10.1002/npr2.12099 (2020). 10.1002/npr2.12099 PMC7722664 32125791

[20] Yamanashi, T. et al. Beta-hydroxybutyrate, an endogenous NLRP3 inflammasome inhibitor, attenuates anxiety-related behavior in a rodent post-traumatic stress disorder model. *Sci. Rep.* **10**, 21629. 10.1038/s41598-020-78410-2 (2020). 33303808 10.1038/s41598-020-78410-2 PMC7728809

[21] Bach, A. & Babayan, V. Medium-chain triglycerides: an update. *Am. J. Clin. Nutr.* **36**, 950–962. 10.1093/ajcn/36.5.950 (1982). 6814231 10.1093/ajcn/36.5.950

[22] Babayan, V. K. Medium chain triglycerides and structured lipids. *Lipids* **22**, 417–420. 10.1007/BF02537271 (1987). 3112486 10.1007/BF02537271

[23] Bodkowski, R. et al. Lipid complex effect on fatty acid profile and chemical composition of cow milk and cheese. *J. Dairy Sci.* **99**, 57–67. 10.3168/jds.2015-9321 (2016). 26506539 10.3168/jds.2015-9321

[24] Traul, K. A., Driedger, A., Ingle, D. L. & Nakhasi, D. Review of the toxicologic properties of medium-chain triglycerides. *Food Chem. Toxicol.* **38**, 79–98. 10.1016/s0278-6915(99)00106-4 (2000). 10685018 10.1016/s0278-6915(99)00106-4

[25] Miyagawa, Y. et al. Intake of medium-chain fatty acids induces myocardial oxidative stress and atrophy. *Lipids Health Dis.* **17**, 258. 10.1186/s12944-018-0908-0 (2018). 30447697 10.1186/s12944-018-0908-0 PMC6240279

[26] Nguyen, P. et al. Liver lipid metabolism. *Anim. Physiol. Nutr.* **92**, 272–283. 10.1111/j.1439-0396.2007.00752.x (2008). 10.1111/j.1439-0396.2007.00752.x 18477307

[27] Shcherbakova, K., Schwarz, A., Apryatin, S., Karpenko, M. & Trofimov, A. Supplementation of Regular Diet With Medium-Chain Triglycerides for Procognitive Effects: A Narrative Review. *Front. Nutr.* **9**, 934497. 10.3389/fnut.2022.934497 (2022). 35911092 10.3389/fnut.2022.934497 PMC9334743

[28] Miura, A. et al. Medium-Chain Triglyceride Administration Induces Antidepressant Effects in Animal Models by Increasing Beta-Hydroxybutyrate Levels. *Yonago Acta Med.* **68**, 58–67. 10.33160/yam.2025.02.007 (2025). 39968118 10.33160/yam.2025.02.007 PMC11831044

[29] Iwata, M. et al. Psychological Stress Activates the Inflammasome via Release of Adenosine Triphosphate and Stimulation of the Purinergic Type 2X7 Receptor. *Biol. Psychiatry*. **80**, 12–22. 10.1016/j.biopsych.2015.11.026 (2016). 26831917 10.1016/j.biopsych.2015.11.026

[30] Ari, C. et al. Exogenous Ketone Supplements Reduce Anxiety-Related Behavior in Sprague-Dawley and Wistar Albino Glaxo/Rijswijk Rats. *Front. Mol. Neurosci.* **9**10.3389/fnmol.2016.00137 (2016). 10.3389/fnmol.2016.00137 PMC5138218 27999529

[31] Edwards, M. G. P., Furuholmen-Jenssen, T., Søegaard, E. G. I., Thapa, S. B. & Andersen, J. R. Exploring diet-induced ketosis with exogenous ketone supplementation as a potential intervention in post-traumatic stress disorder: a feasibility study. *Front. Nutr.* **11**, 1406366. 10.3389/fnut.2024.1406366 (2024). 39588043 10.3389/fnut.2024.1406366 PMC11586679

[32] Mojtabavi, H., Saghazadeh, A., Van Den Heuvel, L., Bucker, J. & Rezaei, N. Peripheral blood levels of brain-derived neurotrophic factor in patients with post-traumatic stress disorder (PTSD): A systematic review and meta-analysis. *PLoS ONE*. **15**, e0241928. 10.1371/journal.pone.0241928 (2020). 33152026 10.1371/journal.pone.0241928 PMC7644072

[33] Linz, R. et al. Acute psychosocial stress increases serum BDNF levels: an antagonistic relation to cortisol but no group differences after mental training. *Neuropsychopharmacol* **44**, 1797–1804. 10.1038/s41386-019-0391-y (2019). 10.1038/s41386-019-0391-y PMC6785147 30991416

[34] Tsukinoki, K. et al. Submandibular Glands Contribute to Increases in Plasma BDNF Levels. *J. Dent. Res.* **86**, 260–264. 10.1177/154405910708600312 (2007). 17314259 10.1177/154405910708600312

[35] Saruta, J. et al. Chronic stress affects the expression of brain-derived neurotrophic factor in rat salivary glands. *Stress* **13**, 53–60. 10.3109/10253890902875167 (2010). 19658028 10.3109/10253890902875167

[36] Hauck, S. et al. Serum levels of brain-derived neurotrophic factor in acute and posttraumatic stress disorder: a case report study. *Rev. Bras. Psiquiatr.* **31**, 48–51. 10.1590/s1516-44462009000100012 (2009). 19506776 10.1590/s1516-44462009000100012

[37] Hauck, S. et al. Serum brain-derived neurotrophic factor in patients with trauma psychopathology. *Prog. Neuropsychopharmacol. Biol. Psychiatry*. **34**, 459–462. 10.1016/j.pnpbp.2010.01.010 (2010). 20097247 10.1016/j.pnpbp.2010.01.010

[38] Matsuoka, Y., Nishi, D., Noguchi, H., Kim, Y. & Hashimoto, K. Longitudinal Changes in Serum Brain-Derived Neurotrophic Factor in Accident Survivors with Posttraumatic Stress Disorder. *Neuropsychobiology* **68**, 44–50. 10.1159/000350950 (2013). 23774996 10.1159/000350950

[39] Chang, S. H. et al. BDNF Protein and BDNF mRNA Expression of the Medial Prefrontal Cortex, Amygdala, and Hippocampus during Situational Reminder in the PTSD Animal Model. *Behav. Neurol.* **2021**, 1–13. 10.1155/2021/6657716 (2021). 10.1155/2021/6657716 PMC7964114 33763156

[40] Bali, A. & Jaggi, A. S. Electric foot shock stress: a useful tool in neuropsychiatric studies. *Rev. Neurosci.* **26**, 655–677. 10.1515/revneuro-2015-0015 (2015). 26167978 10.1515/revneuro-2015-0015

[41] Li, S. H. & Graham, B. M. Why are women so vulnerable to anxiety, trauma-related and stress-related disorders? The potential role of sex hormones. *Lancet Psychiatry*. **4**, 73–82. 10.1016/S2215-0366(16)30358-3 (2017). 27856395 10.1016/S2215-0366(16)30358-3

[42] Diniz, C. R. A. F., Casarotto, P. C., Resstel, L. & Joca, S. R. L. Beyond good and evil: A putative continuum-sorting hypothesis for the functional role of proBDNF/BDNF-propeptide/mBDNF in antidepressant treatment. *Neurosci. Biobehavioral Reviews*. **90**, 70–83. 10.1016/j.neubiorev.2018.04.001 (2018). 10.1016/j.neubiorev.2018.04.001 29626490

[43] Rojas-Morales, P., Tapia, E. & Pedraza-Chaverri, J. β-Hydroxybutyrate: A signaling metabolite in starvation response? *Cell. Signal.* **28**, 917–923. 10.1016/j.cellsig.2016.04.005 (2016). 27083590 10.1016/j.cellsig.2016.04.005

[44] Shimazu, T. et al. Suppression of Oxidative Stress by β-Hydroxybutyrate, an Endogenous Histone Deacetylase Inhibitor. *Science* **339**, 211–214. 10.1126/science.1227166 (2013). 23223453 10.1126/science.1227166 PMC3735349

[45] Llorente-Folch, I., Düssmann, H., Watters, O., Connolly, N. M. C. & Prehn, J. H. M. Ketone body β-hydroxybutyrate (BHB) preserves mitochondrial bioenergetics. *Sci. Rep.* **13** (1), 19664. 10.1038/s41598-023-46776-8 (2023). Published 2023 Nov 11. 37952048 10.1038/s41598-023-46776-8 PMC10640643

[46] Yen, H., Lai, W., Lin, C. & Chiang, S. Medium-chain triglyceride as an alternative of in‐feed colistin sulfate to improve growth performance and intestinal microbial environment in newly weaned pigs. *Anim. Sci. J.* **86**, 99–104. 10.1111/asj.12248 (2015). 25039368 10.1111/asj.12248

[47] Liberzon, I., Krstov, M. & Young, E. A. Stress-restress: Effects on ACTH and fast feedback. *Psychoneuroendocrinology* **22**, 443–453. 10.1016/s0306-4530(97)00044-9 (1997). 9364622 10.1016/s0306-4530(97)00044-9

[48] Souza, R. R., Noble, L. J. & McIntyre, C. K. Using the Single Prolonged Stress Model to Examine the Pathophysiology of PTSD. *Front. Pharmacol.* **8**, 615. 10.3389/fphar.2017.00615 (2017). 28955225 10.3389/fphar.2017.00615 PMC5600994

[49] Rudolfová, V. et al. Inter-individual differences in laboratory rats as revealed by three behavioural tasks. *Sci. Rep.* **12**, 9361. 10.1038/s41598-022-13288-w (2022). 35672428 10.1038/s41598-022-13288-w PMC9174278

[50] Cohen, H., Geva, A. B., Matar, M. A., Zohar, J. & Kaplan, Z. Post-traumatic stress behavioural responses in inbred mouse strains: can genetic predisposition explain phenotypic vulnerability? *Int. J. Neuropsychopharm*. **11**10.1017/S1461145707007912 (2008). 10.1017/S1461145707007912 17655807

[51] Kanda, Y. Investigation of the freely available easy-to-use software ‘EZR’ for medical statistics. *Bone Marrow Transpl.* **48**, 452–458. 10.1038/bmt.2012.244 (2013). 10.1038/bmt.2012.244 PMC3590441 23208313

