# Supplementary material for: Oral administration of MCT oil reduces anxiety-like behavior and is associated with differences in serum BDNF responses in a rat model of PTSD
Source: Sci Rep. 2026 Apr 6;16:16497. doi: 10.1038/s41598-026-46116-6 (PMC13216621; doi:10.1038/s41598-026-46116-6)
Supplement: Supplementary file 1 — Supplementary Material 1 [file 41598_2026_46116_MOESM1_ESM.docx]

**Supplementary Information**

**Title:** Oral administration of MCT oil reduces anxiety-like behavior and is associated with differences in serum BDNF responses in a rat model of PTSD

**Author:** Daisuke Yoshioka^1^, Takehiko Yamanashi^1^, Koji Komatsu^1^, Naofumi Kajitani^1^, Chika Ushida^1^, Miyu Matsumi^1^, Moyu Nakamoto^1^, Kaori Adachi^2^, Ryoichi Matsuo^1^, Akihiko Miura^1^, Tsuyoshi Nishiguchi^1^, and Masaaki Iwata^1^

^1^ Department of Neuropsychiatry, Faculty of Medicine, Tottori University, Tottori, Japan

^2^ Organization for Research Initiative and Promotion, Tottori University, Tottori, Japan

***Corresponding author:** Takehiko Yamanashi MD, PhD

Department of Neuropsychiatry, Faculty of Medicine, Tottori University, Tottori, Japan

**Tel:** +81-859-38-6547

**E-mail:** [yamatake@tottori-u.ac.jp](mailto:yamatake@tottori-u.ac.jp)

**Supplementary Figure 1. Effects of SPS exposure and MCT oil on time spent in the**
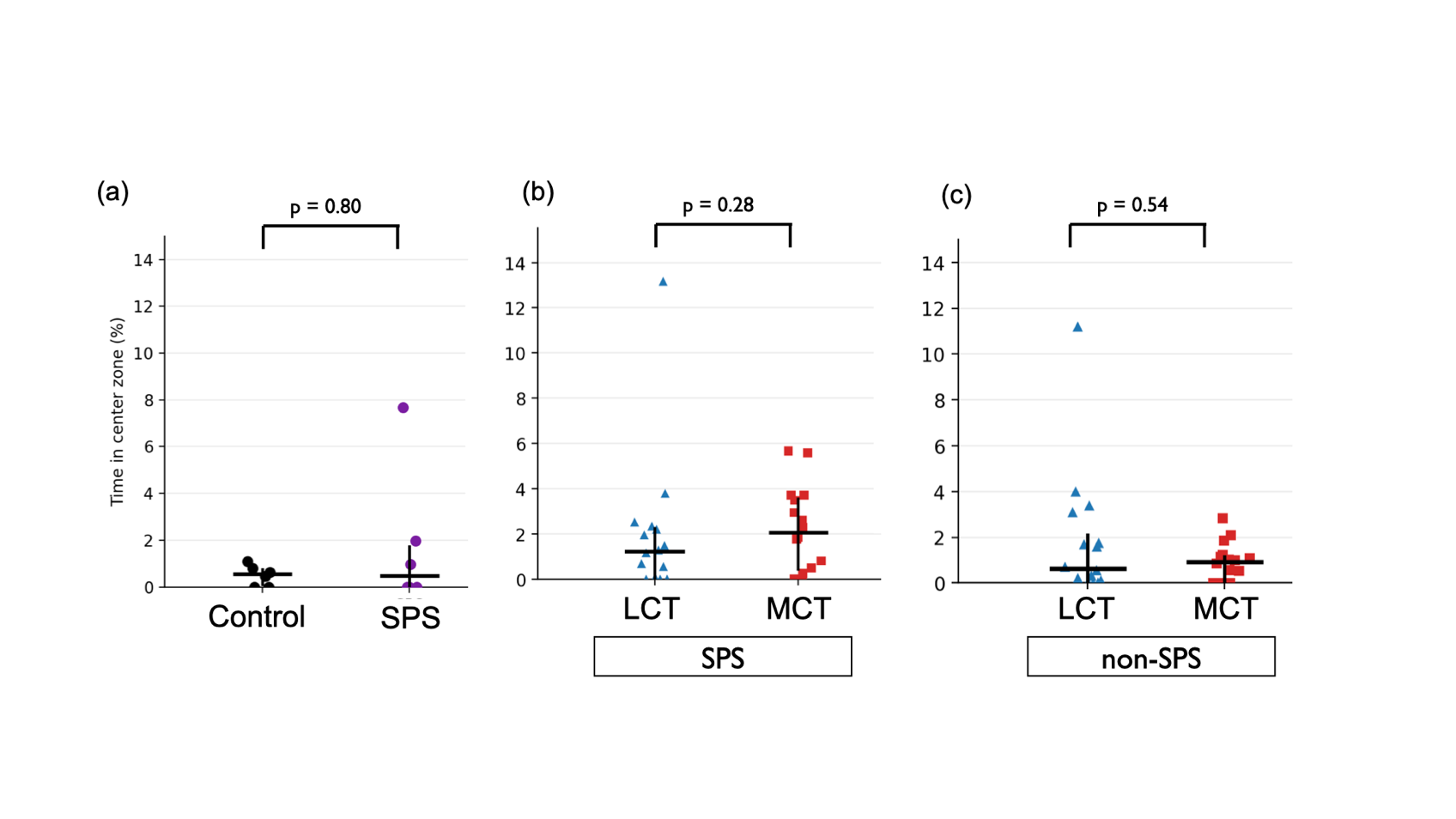
**center zone of the OFT**

Time spent in the center zone of the OFT was analyzed to evaluate the effects of SPS exposure and oral MCT oil administration.

(a) Time spent in the center zone of the OFT did not differ between control and SPS rats (n = 6 per group; median [IQR]; Control: 0.54 [0.11–0.76], SPS: 0.49 [0.00–1.73], p = 0.80).

(b) Oral MCT oil did not significantly alter time spent in the center zone of the OFT in SPS rats (n = 16 per group; median [IQR]; SPS + LCT: 1.22 [0.05–2.24], SPS + MCT: 2.06 [0.42–3.54], p = 0.28).

(c) Oral MCT oil alone did not significantly alter time spent in the center zone of the OFT in non-SPS rats (n = 16 per group; median [IQR]; non-SPS + LCT: 0.64 [0.10–2.10], non-SPS + MCT: 0.93 [0.00–1.17], p = 0.54).

OFT, open field test; MCT, medium-chain triglyceride; LCT, long-chain triglyceride; IQR, interquartile range.

**
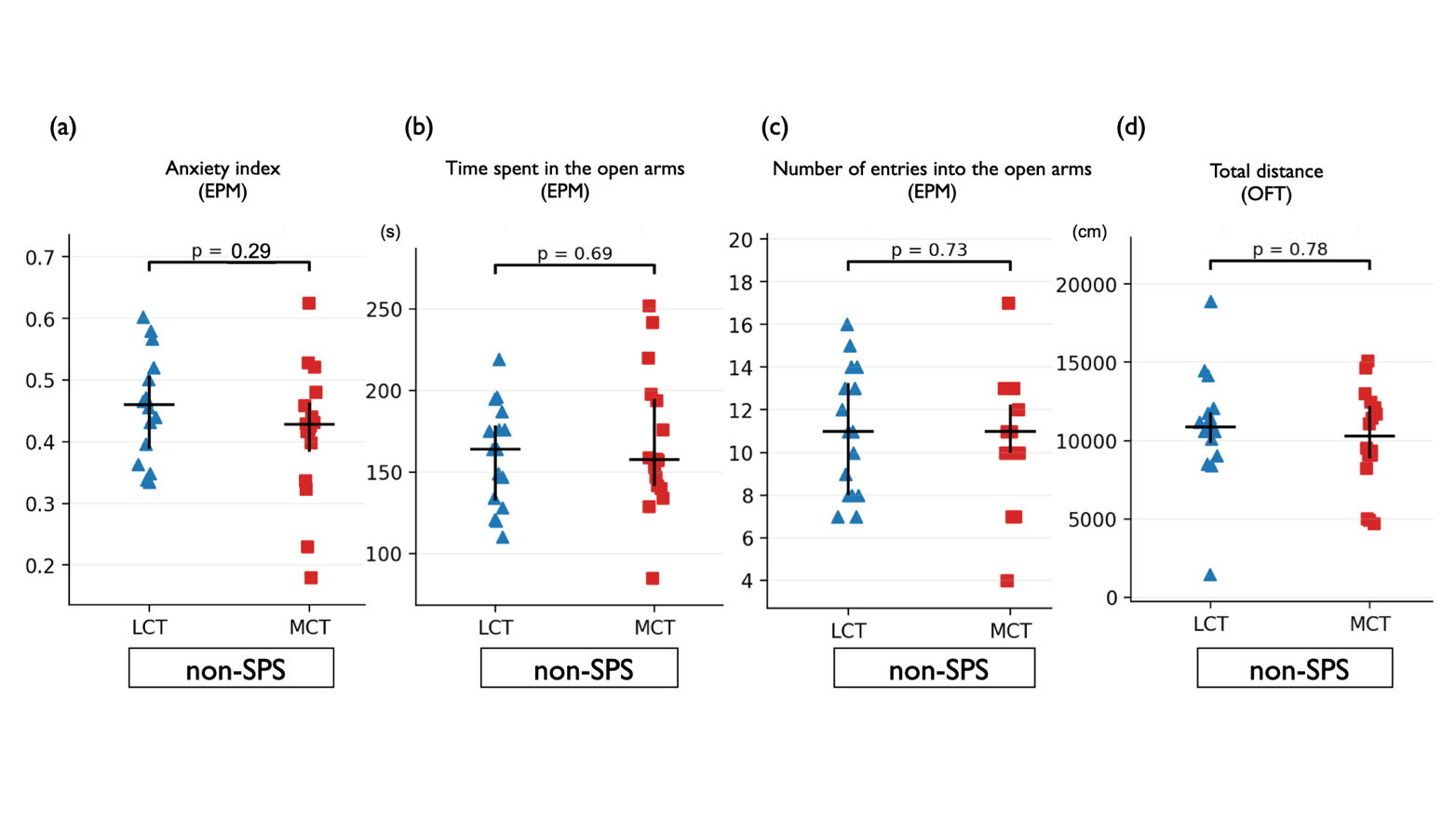
Supplementary Figure 2. MCT alone did not alter anxiety-like behavior or locomotor activity**

Rats without SPS exposure were orally administered 0.8 mL of either MCT or LCT oil daily for 2 weeks. Anxiety-like behavior was evaluated using the EPM, and locomotor activity was assessed using the OFT.

(a) Anxiety index: MCT administration did not alter the anxiety index (median [IQR]; LCT: 0.46 [0.39–0.51], MCT: 0.43 [0.38–0.46], p = 0.29).

(b) Time spent in open arms: MCT administration did not affect the time spent in the open arms (LCT: 164.0 [132.5–178.8], MCT: 157.5 [141.5–195.0], p = 0.69).

(c) Number of open-arm entries: MCT administration did not change the number of open-arm entries (LCT: 11.0 [8.0–13.3], MCT: 11.0 [10.0–12.3], p = 0.73).

(d) Total distance traveled in the OFT: No significant difference was observed between groups (LCT: 10,896.3 [9,856.5–11,830.1], MCT: 10,315.2 [8,864.0–12,204.2], p = 0.78).

These results indicate that oral MCT oil administration alone does not affect anxiety-like behavior or locomotor activity in non-stressed rats. Data are presented as scatter plots showing the median and IQR. The Mann–Whitney U test was used for statistical comparisons. Time variables are expressed in seconds, and distance is expressed in centimeters. Sample size: n = 16 per group.

EPM, elevated plus maze; OFT, open field test; MCT, medium-chain triglyceride; LCT, long-chain triglyceride; IQR, interquartile range.

**Supplementary Figure 3. Time course of body weight following oral MCT oil administration
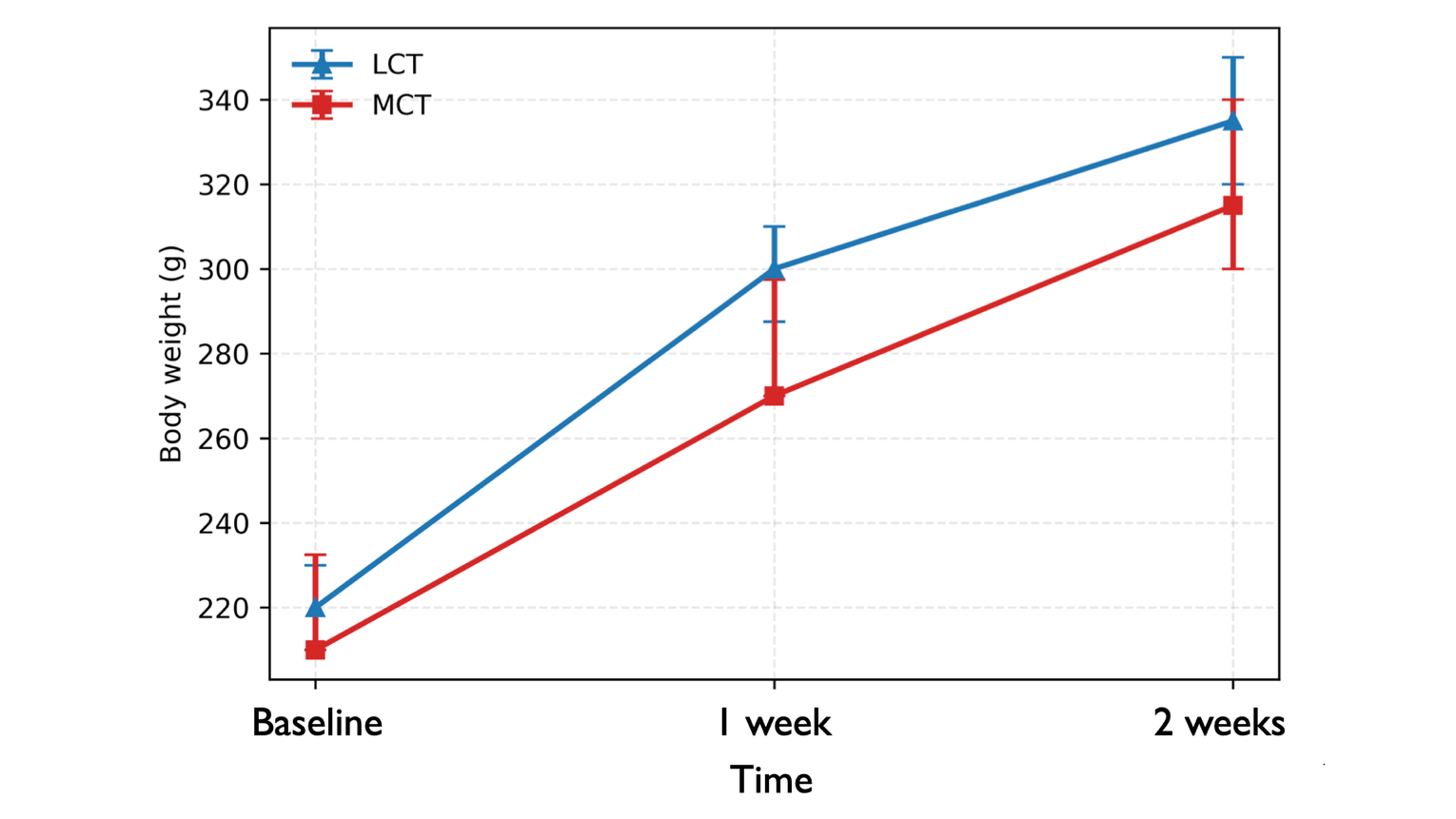
**

Rats received daily oral administration of MCT or LCT oil, and body weight was measured at baseline, 1 week, and 2 weeks after the start of treatment. Body weight increased over time in both groups, and no significant differences were observed between the MCT and LCT groups at either 1 week (median [IQR]; LCT: 300 [287.5–310] g, MCT: 270 [270–297.5] g, p = 0.135) or 2 weeks (LCT: 335 [320–350] g, MCT: 315 [300–340] g, p = 0.196). These results indicate that oral MCT oil administration did not significantly affect body weight compared with LCT oil during the experimental period.

Data are shown as line plots indicating the median and IQR. The Mann–Whitney U test was used for statistical comparisons. Sample size: n = 16 rats per group.

MCT, medium-chain triglyceride; LCT, long-chain triglyceride; IQR, interquartile range.

**
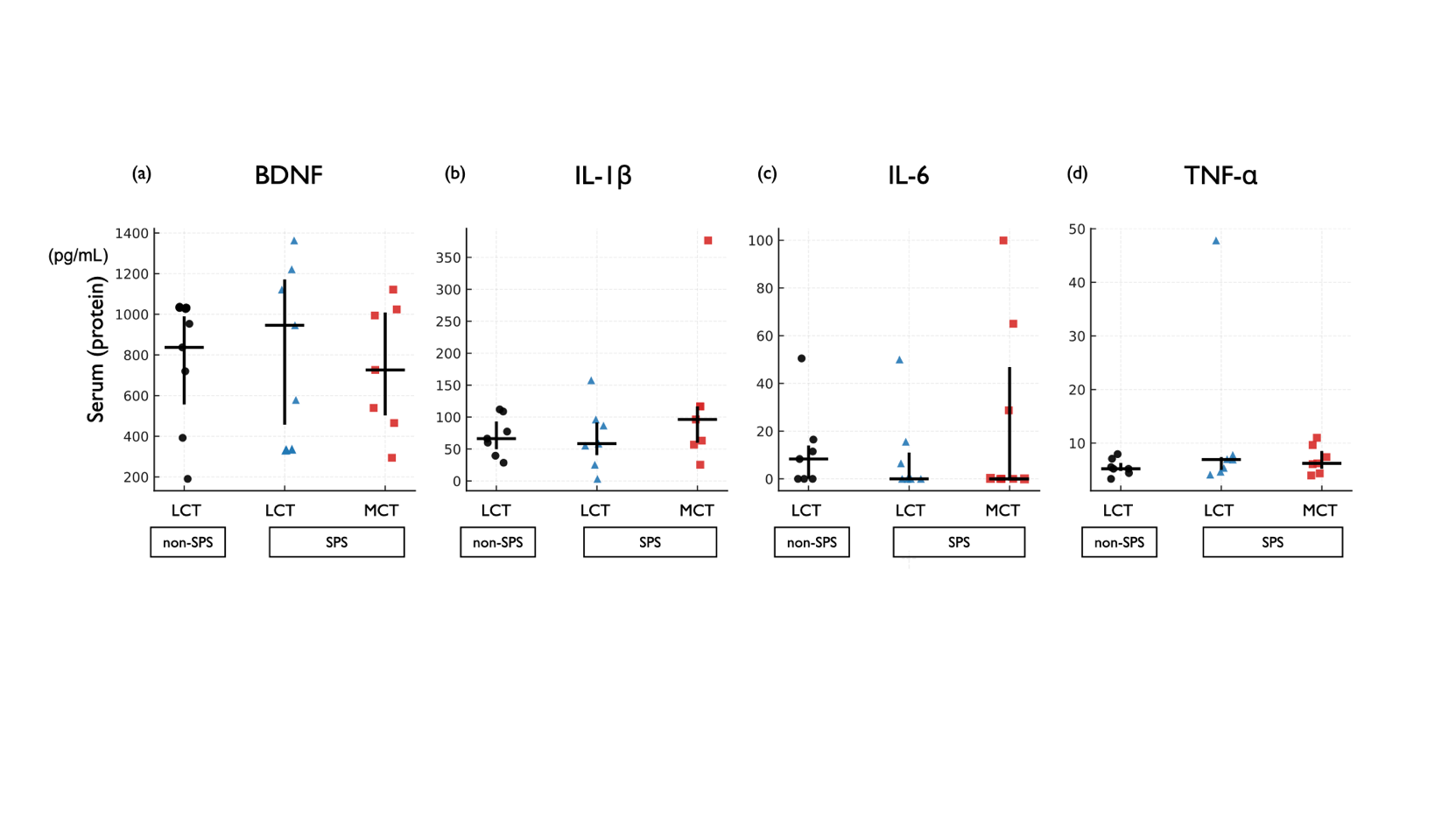
Supplementary Figure 4. Effects of MCT oil on baseline serum BDNF and cytokine levels in SPS rats**

Serum BDNF and cytokine levels were measured after 2 weeks of daily oral MCT or LCT oil administration to non-SPS and SPS rats.

(a) Serum BDNF (median [IQR]; non-SPS + LCT: 837.17 [556.08–989.96], SPS + LCT: 946.12 [457.12–1171.44], SPS + MCT: 726.28 [502.24–1008.98]). p = 1.00 for all pairwise comparisons.

(b) Serum IL-1β (median [IQR]; non-SPS + LCT: 66.28 [49.75–93.05], SPS + LCT: 58.41 [40.30–91.48], SPS + MCT: 96.20 [59.98–116.67]); p = 0.92 for all pairwise comparisons.

(c) Serum IL-6 (median [IQR]; non-SPS + LCT: 8.30 [0.00–13.97], SPS + LCT: 0.00 [0.00–11.02], SPS + MCT: 0.00 [0.00–46.85]); p = 1.00 for all pairwise comparisons.

(d) Serum TNF-α (median [IQR]; non-SPS + LCT: 5.21 [4.82–6.30], SPS + LCT: 6.93 [5.01–7.40], SPS + MCT: 6.23 [5.21–8.53]); p = 1.00 for all pairwise comparisons.

Data are presented as scatter plots showing the median and IQR.

Mann–Whitney U tests were used for pairwise comparisons with Holm-adjusted p-values. Serum BDNF and cytokine levels are expressed in pg/mL as measured by ELISA. Sample size: n = 7 rats per group.

BDNF, brain-derived neurotrophic factor; IL, interleukin; TNF, tumor necrosis factor; SPS, single prolonged stress; LCT, long-chain triglyceride; MCT, medium-chain triglyceride; IQR, interquartile range.

**Supplementary Figure 5. Effects of MCT oil on cytokine responses to acute restraint stress in SPS rats**


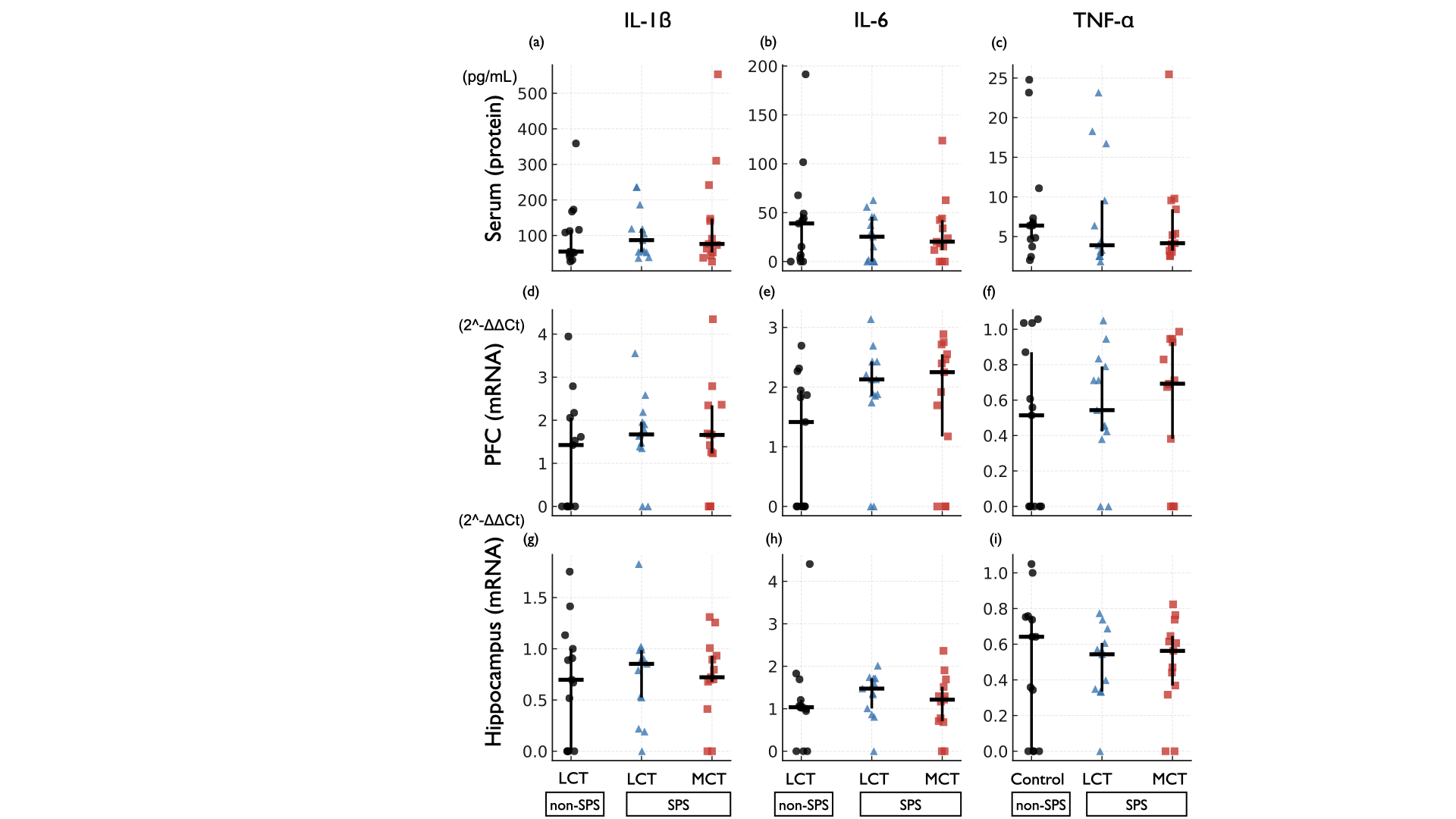


Rats were subjected to 1-h acute restraint stress before euthanasia, and serum and brain samples were collected for cytokine analyses.

(a) Serum IL-1β (median [IQR]; non-SPS + LCT: 54.67 [48.22–115.92], SPS + LCT: 86.91 [53.59–119.15], SPS + MCT: 76.16 [52.52–147.09]); p = 1.00 for all pairwise comparisons.

(b) Serum IL-6 (median [IQR]; non-SPS + LCT: 38.95 [3.36–49.12], SPS + LCT: 25.39 [0.00–45.73], SPS + MCT: 20.31 [11.83–42.34]); p = 1.00 for all pairwise comparisons.

(c) Serum TNF-α (median [IQR]; non-SPS + LCT: 6.38 [4.67–7.32], SPS + LCT: 3.90 [2.53–9.55], SPS + MCT: 4.16 [3.21–8.44]); p = 1.00 for all pairwise comparisons.

(d) PFC IL-1β (median [IQR]; non-SPS + LCT: 1.02 [0.81–1.33], SPS + LCT: 0.95 [0.73–1.24], SPS + MCT: 0.88 [0.70–1.15]); p = 0.97 for all pairwise comparisons.

(e) PFC IL-6 (median [IQR]; non-SPS + LCT: 0.65 [0.45–1.00], SPS + LCT: 1.43 [1.10–1.44], SPS + MCT: 1.20 [0.98–1.63]). Pairwise comparisons: non-SPS + LCT vs. SPS + LCT, p = 0.24; non-SPS + LCT vs. SPS + MCT, p = 0.24; SPS + LCT vs. SPS + MCT, p = 0.84.

(f) PFC TNF-α (median [IQR]; non-SPS + LCT: 1.00 [0.84–1.32], SPS + LCT: 0.86 [0.79–1.04], SPS + MCT: 0.78 [0.75–1.11]); p = 1.00 for all pairwise comparisons.

(g) Hippocampal IL-1β (median [IQR]; non-SPS + LCT: 0.00 [0.00–1.18], SPS + LCT: 0.00 [0.00–1.18], SPS + MCT: 0.93 [0.33–1.17]); p = 1.00 for all pairwise comparisons.

(h) Hippocampal IL-6 (median [IQR]; non-SPS + LCT: 0.00 [0.00–1.18], SPS + LCT: 0.00 [0.00–1.18], SPS + MCT: 0.93 [0.33–1.17]); Pairwise comparisons: non-SPS + LCT vs. SPS + LCT, p = 0.71; non-SPS + LCT vs. SPS + MCT, p = 0.71; SPS + LCT vs. SPS + MCT, p = 1.00.

(i) Hippocampal TNF-α (median [IQR]; non-SPS + LCT: 0.00 [0.00–0.83], SPS + LCT: 0.00 [0.00–0.53], SPS + MCT: 0.26 [0.10–0.36]); p = 1.00 for all pairwise comparisons.

Data are shown as scatter plots (median and IQR). Pairwise comparisons were performed using Mann–Whitney U tests with Holm-adjusted p-values. Serum cytokine concentrations are expressed in pg/mL as measured by ELISA, whereas PFC and hippocampal cytokine values represent relative mRNA expression levels calculated using the 2^-ΔΔCt method. Sample size: n = 13 rats per group.

IL, interleukin; TNF, tumor necrosis factor; PFC, prefrontal cortex; SPS, single prolonged stress; LCT, long-chain triglyceride; MCT, medium-chain triglyceride; IQR, interquartile range.
